# Complete genome sequence of *Mumia* sp. Pv 4-285 isolated from the rhizosphere of *Phyllostachys viridiglaucescens* (Carrière) Rivière & C. Rivière

**DOI:** 10.1128/mra.00822-24

**Published:** 2024-11-29

**Authors:** Anna Kachor, Markiyan Samborskyy, Yuriy Rebets, Oleksandr Gromyko

**Affiliations:** 1 Department of Genetics and Biotechnology, Ivan Franko National University of Lviv, Lviv, Ukraine; 2 Explogen LLC, Lviv, Ukraine; 3 Microbial Culture Collection of Antibiotic Producers, Ivan Franko National University of Lviv, Lviv, Ukraine; The University of Arizona, Tucson, Arizona, USA

**Keywords:** *Mumia* sp., actinomycetes, genome

## Abstract

*Mumia* are one of the rarest representatives of phylum Actinomycetota with only four species known. Here, we report the complete genome sequence of *Mumia* sp. Pv 4-285 isolated from a rhizosphere sample of *Phyllostachys viridiglaucescens*. The genome consists of a 4,649,352 bp circular chromosome with 4,361 protein-coding genes.

## ANNOUNCEMENT

The genus *Mumia* was described by Lee et al. (1) as a member of the family Nocardioidaceae in 2014. *Mumia* is a genus of aerobic Gram-positive bacteria, which comprises four species isolated from different environmental samples (2).

*Mumia* sp. Pv 4-285 was isolated from a rhizospheric soil collected from *Phyllostachys viridiglaucescens* growing in the Nikitsky Botanical Garden (Crimea, Ukraine). The isolate was obtained by direct inoculation of a homogenate of rhizospheric soil in sterile water on HVA medium and incubated at 28°C for 24 days (3). For total DNA extraction, a single colony of Pv 4-285 was picked and cultured in liquid ISP2 medium at 30° C for 72 h. Genomic DNA was extracted using the NucleoSpin Microbial DNA kit (Macherey-Nagel, Germany) following the manufacturer’s protocol. The same genomic DNA extraction was used for both Illumina and Nanopore library creation. The isolate was identified by Sanger sequencing of the 16S rRNA gene (3). Strain identity was confirmed (98,96% identity) for *Mumia* sp. Pv 4-285 (ON237368) as *Mumia xiangluensis* NEAU-KD (NR_146673). To obtain the complete genome sequence, the combination of Illumina and Oxford Nanopore Technologies (ONT) sequencing platforms was applied. Illumina genomic shotgun libraries were prepared using a TruSeq DNA PCR-Free Kit and sequenced on the Illumina Novaseq 6000 system in 2 × 150 bps read mode. For long reads sequencing, a DNA library was prepared using a ligation sequencing kit (SQK-LSK110) (ONT, UK) and sequenced on a Flongle flow-cell (R9.4.1). The ONT raw sequence data were basecalled using Guppy v5.0.11 with dna_r9.4.1_450bps_sup model (4) and adaptors removal was done with Porechop (5). The reads QC for both Illumina and ONT data sets was performed using FastQC (6). Sequencing generated a total of 11,508,586 short reads and 831,130 long reads (*N_50_* 2520 bps). Illumina data were assembled with Newbler v3.0 (7), and ONT data were assembled with Flye v.2.9 b1774 (8). Overlapping ends of the circular genome were trimmed by the Flye assembler. The numbering of genome consensus positions of circular genome was started from the beginning of the *dnaA* gene, found using tblastn on complement strand. Consensus was rotated using Artemis v.16.0.11. The ONT assembly consensus sequence polishing (three rounds) was performed with Medaka v.1.4.4 (9), followed by polishing of ONT assembly with Illumina data using Pilon v.1.24 (10). Default parameters were used for all software except where otherwise noted. Genome annotation was performed with NCBI Prokaryotic Genome Annotation Pipeline version 6.7 (11).

The genome was assembled into a single scaffold 4,649,352 bp in length and G + C content of 70.5%, representing the chromosome of Pv 4-285. The coverage of the genome was 153×. The genome contains 4,361 protein-coding genes, 46 tRNA and 9 rRNA genes, 3 non-coding RNAs, and 12 pseudogenes. The genome analysis with antiSMASH v7.1.0 (12) revealed the presence of five putative secondary metabolites biosynthetic gene clusters. Also, the Type Strain Genome Server genome-based phylogeny analysis (13) revealed that Pv 4-285 could be a new species of the *Mumia* genus that prompts its deep physiological and biochemical studies.

## Data Availability

The genome sequence of *Mumia* sp. Pv 4-285 is available in NCBI GenBank under genome accession number CP162023, BioProject accession number PRJNA1132915, and BioSample accession number SAMN42361281. The raw sequence data are available under Sequence Read Archive (SRA) accession numbers SRR29787219 (Illumina) and SRR29821702 (ONT).
